# Non-operative Management of Uncomplicated Appendicitis: A Review of Indications, Safety, and Clinical Algorithms

**DOI:** 10.7759/cureus.105154

**Published:** 2026-03-13

**Authors:** Mohamed Baana, Meghana Singh, Moses Banfa, Laraib Tariq, Tomas Antoni Barbaroux, Faryal Ali, Ahmad Mahmood, Juan J Prieto, Shaikha Alhajeri, Joy W Youssef, Sudharsan Ramapriya

**Affiliations:** 1 Emergency Medicine, The Hillingdon Hospital, London, GBR; 2 Medicine and Surgery, Umm Al Quwain Hospital, Emirates Health Services, Ajman, ARE; 3 General Surgery, Community First Medical Center, Chiacgo, USA; 4 Emergency Medicine, Hull Royal Infirmary, Kingston upon Hull, GBR; 5 General Internal Medicine, Northampton General Hospital, Northampton, GBR; 6 General Medicine, Anahuac University Northern Campus, Mexico, MEX; 7 General Surgery, Pakistan Institute of Medical Sciences, Islamabad, PAK; 8 General Surgery, Xinjiang Medical University, Urumqi, CHN; 9 Internal Medicine, Universidad Autonoma de Guadalajara, Zapopan, MEX; 10 Internal Medicine, Mubarak Al-Kabeer Hospital, Kuwait City, KWT; 11 Medicine, Cairo University, Cairo, EGY; 12 Internal Medicine, Kasturba Medical College Manipal, Manipal, IND

**Keywords:** antibiotic therapy, clinical algorithms, cost-effectiveness, non-operative management, patient selection, uncomplicated appendicitis

## Abstract

Acute uncomplicated appendicitis has traditionally been managed with appendectomy, yet accumulating evidence supports antibiotic-only treatment as a safe alternative in carefully selected patients. This narrative review synthesizes current data on the indications, safety, treatment protocols and clinical algorithms for non-operative management of uncomplicated appendicitis in adults and children. We summarise key randomised trials, systematic reviews and guidelines evaluating antibiotic regimens, imaging-based definitions of uncomplicated disease, clinical scoring systems, and predictors of treatment failure such as appendicolith, fever, raised inflammatory markers and increased appendiceal diameter. Short-term outcomes consistently demonstrate high initial success rates, low rates of severe adverse events and comparable quality of life to surgery, while long-term data highlight a clinically acceptable recurrence risk in most settings. We also discuss special populations, including paediatric and pregnant patients, and review economic analyses comparing operative and conservative strategies. Finally, we propose practical, evidence-informed algorithms to guide shared decision-making and safe implementation of non-operative care.

## Introduction and background

Acute appendicitis remains one of the most important causes of emergency abdominal surgery worldwide, with appendectomy being regarded as the most definitive treatment [1]. Over time, with more emphasis on the past decade, the interest in non-operative management (NOM) using antibiotics has become much more popular for patients with uncomplicated appendicitis. Furthermore, with the development and improvement of high-quality imaging, randomised trials as well as a more in-depth understanding of the various pathologies of the appendix have all helped transition to this idea [2].

The evidence for antibiotic-only therapy was first solidified by the Appendicitis Acuta (APPAC) randomised control trial, in which it was demonstrated that 73% of patients with CT-confirmed uncomplicated appendicitis had surgery avoided within one year when treated with antibiotics [3]. In the five-year follow-up, a cumulative 39.1% appendectomy rate was observed, without any increase in perforation or severe complications among those who required surgery. Overall, complication rates were lower in the antibiotic group (6.5%) than in the surgical group (24.4%) [4].

The evidence base was further strengthened by the CODA trial (Comparing Outcomes of Antibiotic Drugs and Appendectomy). The trial demonstrated the non-inferiority of antibiotic therapy compared with appendectomy at 30 days [5]. This finding established that the use of antibiotic management is equally effective in resolving acute symptoms in the short-term period. Although 29% of the patients who were randomised into antibiotic therapy underwent appendectomy within 90 days, they experienced lower immediate complication rates, shorter recovery times and reduced healthcare utilisation. Antibiotic therapy was not superior in the sense to surgery in terms of efficacy; it still offered a comparable alternative with various advantages in morbidity and therapy. The finding of an appendicolith on imaging was the most identifiable feature, which was mostly associated with treatment failures and a higher likelihood of some complications [6].

Recent literature further reinforces this shift toward conservative therapy, with multiple systematic reviews and guideline analyses collectively demonstrating that NOM can be both safe and effective for well-selected patients. Across these studies, antibiotic-only treatment has shown high initial success rates, frequently above 90%, with most individuals demonstrating clinical improvement within the first 24-48 hours and severe complications such as sepsis or mortality reported as exceedingly rare [7-9]. This collective evidence challenges the long-held belief that appendicitis is a uniformly progressive disease inevitably leading to perforation, instead supporting a more nuanced understanding in which many cases remain localised and amenable to medical management. Furthermore, global trends indicate increasing adoption of conservative strategies in both adults and paediatric populations, supported by imaging-based patient selection and antibiotic regimens consistent with intra-abdominal infection guidelines. Although variations in hospitalisation duration, monitoring criteria, and definitions of treatment failure persist across studies, the overall findings consistently indicate that, in appropriately selected patients, NOM represents a viable alternative to surgery with comparable short-term outcomes and reduced procedural morbidity [7-9].

As perspectives on the management of acute appendicitis continue to evolve, there is a need for a comprehensive synthesis of current evidence. This review summarises the literature available on antibiotic-only therapy for uncomplicated appendicitis, including pathophysiology, patient selection criteria, treatment regimens, clinical outcomes, and diagnostic algorithms, along with economic and global health implications. By synthesising findings from randomised trials, meta-analyses and consensus guidelines, this review aims to define the role of NOM in modern surgical cases of appendicitis, identify knowledge gaps and provide practical guidance for safe and evidence-based clinical practice.

## Review

Pathophysiology

Understanding the structure and physiological role of the vermiform appendix provides essential context for appreciating the pathophysiological processes that lead to inflammation and acute appendicitis. Islam et al. [10] describe the appendix as a narrow, tubular extension of the caecum, about 6 cm long and 6 mm wide, with variations seen in disease. Its mucosa contains abundant lymphoid tissue that supports mucosal immunity and helps restore normal gut bacteria after gastrointestinal infections. By maintaining microbial balance and regulating local immune responses, the appendix contributes to intestinal homeostasis. When this balance is disturbed through obstruction, infection, or microbial imbalance, an inflammatory response can occur, leading to acute appendicitis [10].

Using 16S rRNA sequencing, studies have shown a marked reduction in commensal bacterial species, particularly Lachnospiraceae, Blautia, and Faecalibacterium. This disruption in the normal gut flora appears to play a significant role in the pathogenesis of acute appendicitis [11]. Obstruction of the appendiceal lumen in children may result from fecaliths, lymphoid hyperplasia, parasitic infections, foreign bodies, neoplasms or intra- and extraluminal masses [12]. A progressive increase in luminal pressure leads to vascular and lymphatic collapse, resulting in ischemia and subsequent bacterial overgrowth. At this stage, acute appendicitis is characterised by inflammation and mural oedema. Obstruction of the appendicular artery, a branch of the ileocolic artery arising from the superior mesenteric artery, further contributes to tissue hypoxia and ischemia [13]. Appendiceal distension then activates visceral afferent fibres via splanchnic sympathetic pathways, producing a diffuse, midline pain referred to the T8-10 dermatomes. Progressive tissue necrosis compromises the integrity of the appendiceal wall, making it thin and vulnerable. This may ultimately result in rupture of the mucosal, muscular, and serosal layers, leading to perforation of the appendix. Acute appendicitis is classified as complicated when there is a peri-appendiceal or distant abscess, perforation, or diffuse peritonitis, all of which require urgent surgical management [14]. 

The decision to use antibiotics alone for the containment and resolution of appendicitis relies heavily on appropriate patient stratification [15]. Not all cases of acute appendicitis respond equally well to conservative therapy, making careful selection essential. A systematic review published by Talan et al. [7] analysed 34 studies where 2944 patients were treated with antibiotics. It showed that antibiotics successfully halted disease progression in over 90% of patients within 72 hours, meaning infection was contained before perforation or abscess formation could occur. The transition between intravenous antibiotics to oral step-downs after initial improvement also further reflected how infection control led to recovery. In carefully selected cases, antibiotics can safely achieve both containment and resolution without the need for surgery [16].

Indications for non-operative management

Distinguishing between uncomplicated and complicated is vital to determine what can be treated conservatively with antibiotics as an alternative to surgery [17]. Clinically uncomplicated or simple appendicitis can be defined as an inflammatory condition of the appendix in which the organ appears inflamed and phlegmonous but lacks evidence of necrosis or perforation, as opposed to complicated appendicitis in which there is focal or transmural necrosis that may progress to perforation [18].

In the multi-institutional nonrandomised controlled study by Minneci et al. [19], uncomplicated appendicitis was characterised radiologically on ultrasound (US), computed tomography (CT), or magnetic resonance imaging (MRI) as an inflamed appendix measuring 1.1 cm or less in diameter, without any associated abscess, fecalith, or phlegmon. US is frequently used as the primary imaging technique for the assessment of acute appendicitis, but its diagnostic reliability can be influenced by the examiner’s level of expertise and individual patient characteristics [20]. In the APPAC III randomised double-blind superiority trial, uncomplicated acute appendicitis was described as an inflamed appendix measuring more than 6 mm in diameter, showing a thickened and contrast-enhanced wall, with some surrounding oedema or a small amount of fluid nearby. Crucially, cases were only considered uncomplicated if there were no features suggestive of a more severe process, such as an appendicolith, perforation, abscess, or a possible tumour [21]. This definition helped distinguish milder presentations from those requiring more urgent surgical intervention.

Calcified deposits within the appendix are referred to as appendicoliths and often lead to complicated appendicitis necessitating immediate surgical intervention [22]. In the study by Kupietzky et al. [23], it was observed that patients with appendicoliths tended to experience recurrence sooner following NOM, yet their overall recurrence rate did not appear to be higher. This finding challenges the long-standing assumption that the presence of an appendicolith inevitably guarantees treatment failure. This was later revisited by Tian [24], highlighting the value of thoughtful counselling and careful risk assessment rather than rigidly adhering to conventional expectations. In practical terms, clinicians can now explain to patients that while an appendicolith may slightly increase the likelihood of an earlier recurrence, the overall risk remains modest, and nonoperative treatment is still a reasonable approach for many. That said, certain groups, such as older individuals or those with weakened immune systems, require more deliberate evaluation, as they seem more susceptible to complications like gangrene or perforation when an appendicolith is present [24].

The effectiveness of NOM in acute appendicitis largely depends on appropriate patient selection and the exclusion of those with gangrenous changes, abscess formation, or diffuse peritonitis [2]. An example of how patients were selected for NOM can be seen in a study [25] during the COVID-19 pandemic. The team developed a specific protocol to identify suitable candidates for conservative treatment of appendicitis, while those who did not meet the criteria proceeded to appendectomy. The inclusion criteria were cautious and aimed to identify patients with mild disease as well as a low risk of complications. Patients considered for NOM typically presented with pain for less than three days, were afebrile on admission and were not pregnant. Laboratory findings included a white blood cell count below 15 × 10⁹/L, and imaging showed an appendix diameter under 1 cm with no evidence of an appendicolith. A history of prior appendicitis or signs of peritonism on examination also excluded patients from conservative treatment. Additional factors associated with successful conservative treatment include a modified Alvarado score below 4 [2].

Evidence increasingly suggests that the management of appendicitis in children requires a distinct approach compared to adults, as the disease may progress differently across age groups. Decker et al. [26] highlighted that adults are more likely to develop simple appendicitis than children, necessitating the need for child-specific research. Their systematic review found both operative and NOM to be safe in paediatric cases, with no reported mortality. However, the variability across studies suggests that further research is needed to clarify how factors such as age, laboratory results, and imaging findings influence the success of NOM. Understanding these predictors would allow clinicians to identify which children are most likely to benefit from conservative management and better guide families through shared decision-making. Similarly, Reis Wolfertstetter et al. [27] supported this patient-tailored approach, recommending hospital admission and close monitoring for children with suspected uncomplicated appendicitis. Their management strategy involved hydration, pain control, and antibiotics when indicated, alongside serial clinical assessments and repeated USs to track progress. This structured observation approach may allow early or mild cases to resolve spontaneously, avoiding unnecessary surgery and its associated risks.

Although robust data in pregnancy are limited, a few case reports have described successful conservative treatment of uncomplicated appendicitis in pregnant women [28-30]. However, one study reported that among patients managed nonoperatively, 15% experienced treatment failure and 12% had recurrence during the same pregnancy [31]. These findings suggest that while NOM may be feasible in carefully selected cases, the risks of failure and recurrence remain clinically significant. The decision to manage appendicitis conservatively during pregnancy should therefore be made cautiously, balancing maternal and fetal safety with the potential to avoid surgery. Overall, the evidence indicates that NOM in pregnancy should not be ruled out entirely, but its use should be reserved for patients with clear evidence of uncomplicated disease and managed under close clinical and imaging surveillance [2,32].

Antibiotic regimens and treatment protocols

With increasing studies and evidence emerging recently, it is being increasingly supported that the NOM of uncomplicated appendicitis can be done with antibiotics alone [21]. Many RCTs [4,19,33] have been done, which have defined the empiric regimens, duration of treatments and protocols guiding us on how uncomplicated appendicitis can be managed nonoperatively. This section aims to summarise antibiotic regimens, treatment durations, and inpatient and outpatient protocols, as well as key trials that have been designed to further evaluate the best course of treatment outside the bounds of appendicectomy.

Most antibiotic regimens follow a certain set of guidelines, known as the Infectious Diseases Society of America (IDSA) and Surgical Infection Society (SIS), for the treatment of mild to moderate intra-abdominal infections [7]. Initial empiric IV options include second or third-generation cephalosporins with metronidazole. Alternatively, single antibiotic regimens involve co-amoxiclav, piperacillin-tazobactam, or ertapenem/meropenem [7]. An example of a hospitalised case regimen includes the use of IV ciprofloxacin 500mg twice daily with metronidazole 500mg every eight hours [33]. These IV antibiotics are typically given for at least 24 hours [33]. Step-down oral therapy is usually started once symptoms improve, the patient tolerates oral intake, and physical findings have resolved [7,34]. Standard oral antibiotics that are used involve fluoroquinolones or advanced generation cephalosporins with metronidazole or co-amoxiclav [7]. An example of an oral regimen commonly used would include ciprofloxacin 500mg twice daily with metronidazole 500mg three times daily; this is used in an outpatient approach, with patients being reviewed seven and 14 days post-discharge [34]. Total antibiotic duration typically averages 7-10 days [33]. Furthermore, shorter and narrower regimens are now increasingly being evaluated to limit antimicrobial resistance and to reduce the hospital stay.

Hospital-based regimens usually begin with a minimum of 24 hours of intravenous antibiotics along with symptomatic care [19,34]. The step down to oral therapy begins once pain and tenderness subsides, and the dietary intake improves, typically within 12-24 hours [19]. The discharge criteria include resolution of symptoms, tolerance of diet, and adequate pain control [34]. The outpatient pathway involves oral antibiotics with a close follow-up; trials are being held to explore the option of oral-only regimens and direct ED discharge [21]. These evolving designs reflect a global shift towards minimal inpatient care.

The Finnish APPAC trial series forms the cornerstone of antibiotic-based management research. The original APPAC trial compared appendicectomy with antibiotics in CT-confirmed uncomplicated appendicitis. Patients in the antibiotic arm received intravenous ertapenem for three days, followed by oral levofloxacin and oral metronidazole three times daily for seven days [35]. It demonstrated the non-inferiority of antibiotic therapy, and long-term follow-up confirmed sustained safety and feasibility [4]. APPAC II compared a seven-day course of oral moxifloxacin with the original IV-to-oral regimen [35]. Success rates were 70.2% (oral only) versus 73.8% (IV to oral); although statistical noninferiority was not proven, clinical outcomes were similar [36]. A secondary analysis of APPAC I and II identified predictors of antibiotic failure as appendiceal diameter > 15mm, fever > 38 degrees Celsius, and C-reactive protein (CRP) > 100 mg/L; this highlighted the importance of patient selection [35].

APPAC III was the first double blind placebo-controlled trial comparing antibiotic therapy (IV ertapenem followed up with oral levofloxacin and metronidazole) with placebo. The need for surgery did not differ significantly between groups, suggesting that some cases may resolve spontaneously [21]. The ongoing APPAC IV trial is testing oral moxifloxacin versus placebo in an outpatient non-inferiority design [37]. If the placebo proves non-inferior, it may redefine uncomplicated appendicitis management by eliminating unnecessary antibiotic use.

Beyond the APPAC studies, the CODA trial compared antibiotics with appendectomy, using IV antibiotics for at least 24 hours followed by oral therapy for 10 days, with flexible inpatient or outpatient management. Antibiotics were chosen per IDSA and SIS guidelines on intra-abdominal infections and yielded noninferior outcomes [5]. Similarly, Minneci et al. reported in children that a seven-day regimen (IV piperacillin-tazobactam with metronidazole, followed up with oral co-amoxiclav or ciprofloxacin with metronidazole) achieved noninferiority to surgery [19]. 

Across the trials, total antibiotic duration typically ranges from 7 to 10 days, with early transition to oral therapy once stable. Oral-only and outpatient regimens are increasingly validated, reflecting a move toward conservative, minimal course management. Long-term outcomes demonstrate comparable safety and recurrence rates to surgery in appropriately selected patients. Ongoing research, particularly APPAC IV, will determine whether antibiotics can ultimately be omitted, shaping future algorithms for the NOM of uncomplicated appendicitis.

Evidence from recent literature

Over the last decade, multiple clinical trials have evaluated the efficacy of NOM for acute appendicitis. This growing interest stems from concerns regarding the potential complications, financial burden and impact on patients’ quality of life associated with surgical treatment. Several studies have focused on pediatric populations, a vulnerable group more prone to postoperative complications.

A prospective, nonrandomised controlled study was conducted in 2020 across 10 children’s hospitals in the Midwest [19]. The primary outcomes assessed were disability days and the success rate of NOM at one year. The authors concluded that antibiotic therapy alone achieved a success rate of 67.1%, with fewer disability days at one year. Among the 1,068 patients aged 7 to 17 years, postoperative complications included infections (1.1%), reoperations (0.6%), readmissions (2.9%), and emergency visits (6.9%). However, loss to follow-up was a limitation, resulting in statistically nonsignificant outcomes. Most caregivers initially expressed discomfort with NOM, which was mitigated through the use of long-acting antibiotics, emergency monitoring, and outpatient follow-up. Although this protocol has demonstrated success in adults, evidence in pediatric populations remains limited.

In contrast, a retrospective cohort study conducted between 2011 and 2020 demonstrated a high failure rate of nonoperative treatment [38]. Among 117,705 children under 19 years of age, 73,544 were diagnosed with nonperforated appendicitis. Of these, 14% were treated nonoperatively, and 20.1% experienced a reencounter within 14 days, defined as early treatment failure. The majority of these failures were attributed to perforated appendicitis at recurrence. Children who received both nonoperative and operative management experienced higher complication rates than those who underwent immediate surgery.

A randomised controlled trial conducted at the Mayo Clinic between 2018 and 2019 included 180 paediatric patients aged 5 to 15 years and compared nonoperative treatment with intravenous and oral antibiotics versus surgical appendectomy [39]. Failure was defined as abscess formation, peri-appendiceal fluid collection on US, need for surgery within 48 hours, or recurrence within six months. The study reported a success rate of 84% for NOM, supporting its consideration as a primary treatment option. However, at five-year follow-up, 46% of patients had undergone appendectomy, and 14% were diagnosed with recurrent appendicitis confirmed histologically.

The APPAC-I randomised controlled trial (2017) compared open appendectomy with a combination antibiotic regimen (IV ertapenem followed by oral levofloxacin and metronidazole) in children aged 7-17 years [4]. The primary outcome was the complication rate at one year. Among 334 patients, 39.1% of those initially treated with antibiotics required appendectomy within five years. Although not powered to study treatment-related complications, most reported complications were infectious, likely due to a higher proportion of open surgeries compared with laparoscopic procedures. The APPAC-II trial (2021) compared oral moxifloxacin with intravenous ertapenem plus oral levofloxacin and found that approximately 70% (207 of 295) of patients avoided surgery, supporting APPAC-I findings [36].

The APPAC-III trial (2022) investigated placebo versus antibiotic treatment to assess the necessity of antibiotics in uncomplicated appendicitis, given rising antibiotic resistance concerns [21]. Due to enrollment challenges, only 66 patients from five hospitals were included. The primary endpoint was successful treatment, defined as discharge within 10 days without appendectomy. Only 7% of the placebo group and 3% of the antibiotic group required appendectomy within 10 days of admission.

The CODA trial (2020), conducted across 25 healthcare centres in the United States, enrolled 1,552 patients randomised to antibiotic therapy or appendectomy [33]. The primary outcome was 30-day health status assessed using the EuroQol-5D score. By 90 days, approximately 29% of patients in the antibiotic group had undergone appendectomy. Severe adverse events occurred in 4% of the antibiotic group (e.g., percutaneous drainage, C. difficile infection) and 3% of the surgical group. The higher complication rate in the antibiotic arm was attributed to the inclusion of patients with severe appendicitis, unlike the APPAC trials, which included only uncomplicated cases. A subsequent cohort analysis of 776 CODA participants evaluated outpatient antibiotic treatment [40]. Patients discharged within 24 hours missed fewer workdays and had fewer emergency visits within seven days. However, the short follow-up duration limited the evaluation of long-term complications.

A 2019 systematic review of 21 studies, including a meta-analysis of 16 studies, assessed the efficacy of NOM versus appendectomy for acute appendicitis in children [41]. The review noted that approximately 60% of patients with appendicoliths in the nonoperative group ultimately required surgery. Similarly, in the APPAC-I trial, patients with CT-confirmed appendicoliths were excluded due to their association with recurrent appendicitis [4]. Some studies also reported higher parental satisfaction and quality of life following appendectomy at one year. Major limitations included heterogeneity across studies, differences in age, inclusion criteria, and antibiotic regimens, resulting in selection bias. The absence of long-term follow-up further limited conclusions regarding recurrence and complications. Another systematic review of randomised controlled trials (2022) similarly supported the efficacy of NOM but found no statistically significant differences in short-term outcomes, though a trend toward fewer complications with longer follow-up was noted [11].

A U.S. cost-effectiveness analysis (2019) compared outpatient and inpatient NOM with surgical management [42]. Outpatient NOM was the most cost-effective overall, while surgery became more cost-effective when complications were considered. A similar 2021 study from Colombia found that laparoscopic appendectomy had the highest net monetary benefit compared with open surgery and NOM [43]. NOM remains a lower-cost alternative in settings lacking surgical expertise or resources, particularly where laparoscopic facilities are unavailable.

Safety and outcomes

The rates of serious adverse events (SAEs), represented by appendiceal perforation, abscess formation, sepsis, or other issues that necessitated hospital-level care, were 0.9 per 100 outpatients as compared to 1.3 per 100 inpatients in the appendicolith group. On the whole, the rates of SAEs were 2.3 (95% CI 0.3-8.2) per 100 outpatients compared to 2.8 (95% CI 0.6-7.9) per 100 inpatients [40].

Likewise, a meta-analysis by de Almeida Leite et al. [11] found similar rates of major side effects between appendectomy and antibiotic groups, whereas the appendicitis recurrence in the antibiotic group attained 18% (RR 1.48; 95% CI 1.26-1.70). Data from Salminen et al. [4] also showed a recurrence of 39.1% at five years, making antibiotics a suitable choice for uncomplicated acute appendicitis, especially where the risk from surgery was higher, or there were resource limitations.

However, new data from a randomised controlled trial conducted by St Peter et al. [44], including children aged 5-16 years, refuted previous conclusions in favour of antibiotic therapy. The study found that the recurrence of appendicitis in the antibiotic group was associated with mild to moderate adverse events when compared to those who underwent appendectomy (RR 4.3; 95% CI 2.1-8.7). The failure of treatment, defined as the need for subsequent appendectomy or recurrence, occurred in 28 of 394 patients (7%) in the appendectomy group, compared to 153 of 452 (34%) in the antibiotic group, constituting an absolute difference of 26.7% (90% CI 22.4-30.9).

Comparing the length of hospital stay, McCutcheon et al. [45] pointed out that the stay of the patients treated with antibiotics was considerably longer (3.2 days vs 2.1 days in appendectomy). St Peter et al. [44] also observed that at 12 months, the median stay in hospital was 1.6 days (IQR 1.0-2.6) for the antibiotic group and 1.0 day (IQR 0.75-1.7) for the appendectomy group.

As for delayed complications, presence of appendicolith seems to play the most important role. Although delayed perforation with recurrence is a rare occurrence in big trials of uncomplicated appendicitis (APPAC and CODA), Sohail et al. [46] pointed out that the patient with appendicoliths, and some additional risk factors such as the elderly, obesity, and hyponatremia, had a significantly increased risk of perforation (16.2%). Therefore, the identification of appendicoliths should lead to careful patient selection and counselling before opting for NOM.

Salminen et al. [4] also found that 85 out of 257 initially treated with antibiotics later required appendectomy due to recurrence. Of these, 76 had uncomplicated appendicitis, two had perforation or abscess with complicated appendicitis, and seven had no appendicitis. The delayed perforation was thus exceptionally low, only 2 of the 85 cases (≈ 2.4).

Considering quality of life (QoL), Sippola et al. [36] showed that beyond seven years, QoL between appendectomy and antibiotic group was equal among patients with uncomplicated appendicitis. However, Herrod et al. [47] reported that long-term (12 months) QoL exceeded after appendectomy, especially where the use of antibiotics failed; meanwhile, QoL in the short-term (30 days) remained equal.

In a study by Selänne et al. [35], predictors in favour of appendectomy included the presence of an appendicolith, an appendiceal diameter ≥ 15 mm, and fever > 38 °C. Conversely, a CRP level < 100 mg/L after 24 hours of antibiotic therapy was strongly associated with successful NOM, with a negative predictive value of 99%. Xu et al. [48] also concluded that while appendectomy offered a higher one-year success rate without recurrence, antibiotic therapy was a better alternative for managing uncomplicated appendicitis, although the risk of treatment failure rises in the presence of appendicoliths.

Imaging and the role of diagnostics

As part of the standard diagnostic assessment required to diagnose and establish the complexity of appendicitis, Imaging modalities such as US, which is widely used across Europe, computed tomography (CT) scans, most widely utilised in the United States and MRI, are frequently used [49]. Once complexity is established, the consideration to treat surgically vs a considered approached with antibiotics and probable surgical intervention can usually be reached. It is worth discussing the shortcomings which exist when utilising US to diagnose acute appendicitis when compared to CT and MRI. US is found to have 32% sensitivity and 93% specificity when compared to CT (94% sensitivity and 94% specificity) and MRI (96% sensitivity and 96% specificity) [49]. Operator expertise is of value in increasing the overall sensitivity when using US and is heavily influenced when utilised by less experienced ultrasoundographers [50].

In children, in addition to the medical history, the utilisation of US should be considered as the first option to ascertain the complexity of appendicitis [51]. A major drawback is that the proficiency of the use of US by the radiographer would determine the accuracy of the investigation, and a negative finding may not necessarily exclude appendicitis. Figures quoted by Teoule et al. in their meta-analysis show a higher sensitivity for children of 96% and a specificity of 100% when compared to lower numbers seen in adults [51].

CT seems to perform significantly better in comparison to US, with 76-100% sensitivities and 83-100% specificities as reported by Teoule et al. [51]. Due to more frequent use of CT in the U.S., compared to the predilection of use of clinical judgement and Ultrasonography in Europe, it has been discovered that the rate of negative appendectomies in the US is quoted with figures around 5% [51], as compared to around 32% in Europe [52]. It is worth noting that currently there is no guidance on an ‘acceptable’ Negative appendectomy rate [52].

In a recent study conducted in 2023, the accuracy and safety profile were highlighted, particularly in children [53]. Data analysis of children diagnosed with uncomplicated appendicitis at three months, six months and nine months [53], with an assessment of appendiceal diameter. An increase in appendiceal diameter > 6cm was associated with recurrence of disease in 17/29 (58.6%) of participants [53]. The study concluded that steady resolution of appendiceal diameter was seen on follow-up US and as mentioned above, a high chance of recurrence in patients with diameters greater than 6cm, proving the importance of US in safety surveillance and forecasting repeated episodes of worsening appendicitis [53].

Scoring systems have been proven to be very effective in confirming uncomplicated status, providing value in the decision to treat operatively vs non-operative/conservative management. The Alvarado Score and the acute inflammatory response (AIR) are the two most often used and the Alvarado score has been researched the most [54]. The Alvarado score takes into consideration certain components namely, migration of pain; anorexia; nausea; tenderness in the right lower quadrant; rebound tenderness; elevated temperature; leucocytosis; shift of white blood cell count to the left, with a total score of 10 [54]. This can be useful to rule in/rule out appendicitis with a score of 5 and above and has the added benefit of classifying patients into groups for consideration for discharge and using operative vs non-operative management [54]. A meta-analysis conducted by Ohle et al. (2016) found that “the Alvarado score discriminated well as an observation/admission criterion (Cut point of 5) by achieving high pooled sensitivity of 99% overall”. When analysing its importance in selecting the patient cohort, that would drop in sensitivity to 82% in men and 86% in women and similar figures were also observed in children [54].

The more recent AIR score, formulated in 2008, contains eight variables: vomiting, right iliac fossa (RIF) pain, rebound tenderness or guarding (graded as light, medium, or strong), temperature >38.5 °C, white cell count (WCC), proportion of neutrophils, CRP, and a left shift in neutrophils. This scoring system has demonstrated better diagnostic value compared with the Alvarado score [2]. The meta-analysis conducted by Di Saverio et al. pointed to a sensitivity of 97% to 92 % for severe appendicitis and similar performance when taking all appendicitis into consideration (93% to 88%, respectively).

The scoring mentioned above would provide different results when assessing appendicitis in children. However, when analysing the paediatric appendicitis score vs the Alvarado score, the Alvarado score was found to be more superior in comparison [2].

Future directions

Future directions in the NOM of acute appendicitis are rapidly evolving, as accumulating evidence invites a more nuanced alternative to automatic appendectomy. Central to this shift is improved patient selection: combining imaging findings (e.g., appendiceal diameter, wall defect, presence of appendicolith) with biomarkers (e.g., CRPs and white blood cells) and clinical scoring systems can help predict which patients may safely avoid surgery [15]. For example, the presence of an appendicolith has been consistently associated with higher failure and recurrence rates after antibiotic-only management. Alongside this, the use of predictive algorithms and decision-support tools is gaining traction as a way to integrate multiple variables into a personalised risk profile [15].

Another major frontier is optimising antibiotic and adjunctive therapy regimens. Recent open-access reviews outline trials comparing shorter vs longer antibiotic courses, fully oral regimens, outpatient-based care, and even microbiome-modulating adjuncts (such as probiotics or localised anti-inflammatory delivery) to reduce hospital stay, antibiotic exposure and long-term recurrence [8,11]. Meanwhile, long-term outcome data remain limited. High-quality registries and large prospective studies are needed to assess recurrence rates beyond 1-2 years, incidence of missed appendiceal neoplasms, and the role of interval appendectomy in specific sub-populations [6]. Finally, embedding shared decision-making into the care pathway is vital: patients should understand the trade-offs, which is recurrence risk vs avoiding surgery and have their preferences considered in the algorithm [6].

## Conclusions

Non-operative antibiotic management is a safe and effective alternative to appendectomy for carefully selected patients with uncomplicated appendicitis, offering comparable short-term outcomes and acceptable recurrence rates. Future practice should focus on refined patient selection, standardised protocols, and shared decision-making to integrate conservative management into routine care without compromising safety.
